# Influenza A(H5N1) Immune Response among Ferrets with Influenza A(H1N1)pdm09 Immunity 

**DOI:** 10.3201/eid3103.241485

**Published:** 2025-03

**Authors:** Valerie Le Sage, Bailee D. Werner, Grace A. Merrbach, Sarah E. Petnuch, Aoife K. O’Connell, Holly C. Simmons, Kevin R. McCarthy, Douglas S. Reed, Louise H. Moncla, Disha Bhavsar, Florian Krammer, Nicholas A. Crossland, Anita K. McElroy, W. Paul Duprex, Seema S. Lakdawala

**Affiliations:** Author affiliations: University of Pittsburgh, Pittsburgh, Pennsylvania, USA (V. Le Sage, B.D. Werner, G.A. Merrbach, S.E. Petnuch, H.C. Simmons, K.R. McCarthy, D.S. Reed, L.H. Moncla, A.K. McElroy, W.P. Duprex); Boston University, Boston, Massachusetts, USA (A.K. O’Connell, N.A. Crossland); Icahn School of Medicine at Mount Sinai, New York, New York, USA (D. Bhavsar, F. Krammer); Medical University of Vienna, Vienna, Austria (F. Krammer); Boston University Chobanian & Avedisian School of Medicine, Boston, Massachusetts, USA (N.A. Crossland); Emory University, Atlanta, Georgia, USA (S.S. Lakdawala)

**Keywords:** Influenza, viruses, respiratory infections, dairy cow, H5N1, A(H1N1)pdm09, immunity, pathogenesis, ferret

## Abstract

The emergence of highly pathogenic avian influenza A(H5N1) virus in dairy cattle herds across the United States in 2024 caused several human infections. Understanding the risk for spillover infections into humans is crucial for protecting public health. We investigated whether immunity from influenza A(H1N1)pdm09 (pH1N1) virus would provide protection from death and severe clinical disease among ferrets intranasally infected with H5N1 virus from dairy cows from the 2024 outbreak. We observed differential tissue tropism among pH1N1-immune ferrets. pH1N1-immune ferrets also had little H5N1 viral dissemination to organs outside the respiratory tract and much less H5N1 virus in nasal secretions and the respiratory tract than naive ferrets. In addition, ferrets with pH1N1 immunity produced antibodies that cross-reacted with H5N1 neuraminidase protein. Taken together, our results suggest that humans with immunity to human seasonal influenza viruses may experience milder disease from the 2024 influenza A(H5N1) virus strain.

In March 2024, an outbreak of highly pathogenic avian influenza A(H5N1) clade 2.3.4.4b virus was identified in dairy cattle herds in Texas, USA, and then spread to >400 herds in >15 states (*1*). That spread emphasized the need to monitor H5N1 clade 2.3.4.4b for pandemic potential. H5N1 clade 2.3.4.4b virus infections of various mammals resulted in severe disease and death, including among foxes, mink, cats, cetaceans, pinnipeds, and cows (*2*,*3*). In early April 2024, a case of human infection was identified in Texas (*4*), and more human H5N1 cases were identified among workers associated with poultry or dairy farms in California, Missouri, Michigan, Colorado, and Washington (*5*). In August 2024, human infections in the United States were characterized by conjunctivitis and mild respiratory symptoms, and most did not require hospitalization (*5*).

Most persons experience their first influenza virus infection by 5 years of age (*6*). Thus, current H5N1 human infections are occurring among persons with prior influenza A virus (IAV) immunity. The reduced disease severity among persons infected with the 2024 H5N1 virus might be driven by immunity to human seasonal influenza viruses. Statistical modeling analysis of known human cases of H5N1 and H7N9 infection indicated that childhood hemagglutinin (HA) imprinting may provide lifelong protection against severe infection and death from those viruses (*7*). Specifically, previous research has suggested that immune imprinting with human seasonal H1N1 or H2N2 influenza viruses would reduce disease severity to H5N1 because H5, H1, and H2 share a similar group 1 HA stalk domain (*7*). Despite the potential effects such immunity could have to reduce H5N1 replication and pathogenesis, risk assessment of the 2024 H5N1 outbreak strain has only been performed in immunologically naive ferrets (*8*). We investigated whether ferrets with H1N1 immunity would experience reduced virus replication and disease severity from dairy cow H5N1 virus. 

## Methods

### Cell Preparation

We obtained MDCK cells and human 293T cells from the American Type Culture Collection (https://www.atcc.org). We maintained the MDCK cells in minimum essential medium and the 293T cells in Dulbecco Modified Eagle Medium. We supplemented both cell media with 10% fetal bovine serum, 2 mmol L-glutamine, 100 U/mL penicillin, and 100 mg/mL streptomycin. We incubated cells at 37°C with 5% carbon dioxide (CO_2_). We obtained human 293F cells Medium (Thermo Fisher Scientific, https://www.fishersci.com) and maintained cells at 37°C with 5%–8% CO_2_ in FreeStyle 293 Expression (Thermo Fisher Scientific) supplemented with 100 U/mL penicillin and 100 mg/mL streptomycin.

### Virus Generation

We generated A/dairy cattle/Texas/24-008749-001/2024(H5N1) (GISAID accession no. EPI_ISL_19014384) virus from 8 plasmid reverse genetics system and propagated in MDCK cells (Appendix). We determined noncoding regions for each segment from consensus alignment of H5N1 strains from the 2.3.4.4b clade viruses. 

### Human Subjects Research and Ethics Statement

**Figure 1:**
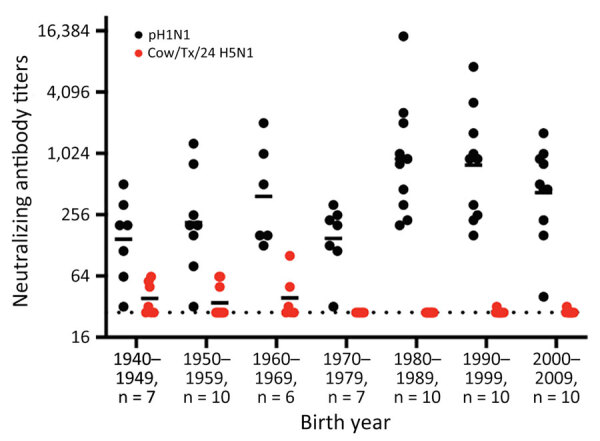
Neutralizing antibody titers in human serum used in a study of influenza A(H5N1) immune response among ferrets with pH1N1 immunity. We used serum samples collected from healthy persons during 2020–2021 with birth years ranging from 1940–2009. We tested serum for neutralizing antibodies against pH1N1 virus and 2024 outbreak virus A/dairy cattle/Texas/24-008749-001/2024(H5N1). Each dot represents the neutralizing antibody titer of a single person to neutralize 100 TCID_50_ of pH1N1 or cow/Tx/24 H5N1 on Madin-Darby canine kidney cells. Solid horizontal lines indicate the geometric mean value each birth decade; dotted line represents the limit of detection for the assay. cow/Tx/24, A/dairy cattle/Texas/24-008749-001/2024(H5N1); pH1N1, influenza A(H1N1)pdm09; TCID_50_, 50% tissue culture infectious dose.

As part of this research, we assessed human serum samples for cross-reactive antibodies to H5N1 virus (Figure 1). The University of Pittsburgh institutional review board approved collection of serum samples from healthy adult donors who provided written informed consent for their samples to be used in infectious disease research (protocol approval no. STUDY20030228). All participants self-reported age, sex, ethnicity, and race.

### Ferret Infections

We screened ferrets before this study to ensure no influenza immunity before they arrived at University of Pittsburgh (Appendix). Using our previously developed preimmune ferret model (*9*,*10*), we infected 5 ferrets with recombinant influenza A(H1N1)pdm09 (pH1N1) virus by using the A/California/07/2009 strain. We infected ferrets either experimentally by intranasal introduction of A/California/07/2009 or naturally by exposure to an experimentally infected ferret in a controlled transmission study conducted at the University of Pittsburgh. 

pH1N1-immune animals then recovered from acquired infections and were housed for 98 days before we infected 5 pH1N1-immune and 5 immunologically naive ferrets with A/dairy cattle/Texas/24-008749-001/2024(H5N1), termed cow/Tx/24 H5N1. We intranasally inoculated all 10 ferrets with 10^4^ 50% tissue culture infectious dose (TCID_50_) cow/Tx/24 H5N1 virus in 500 µL of L-15 media (250 µL in each nostril). We monitored ferrets daily during the postinoculation period and recorded clinical signs, including weight loss, temperature, activity, sneezing, coughing, and nasal discharge, as previously described (*11*). For animals that reached >10% weight loss, we provided urgent care diet cat food 2×/day to entice eating. 

**Figure 2:**
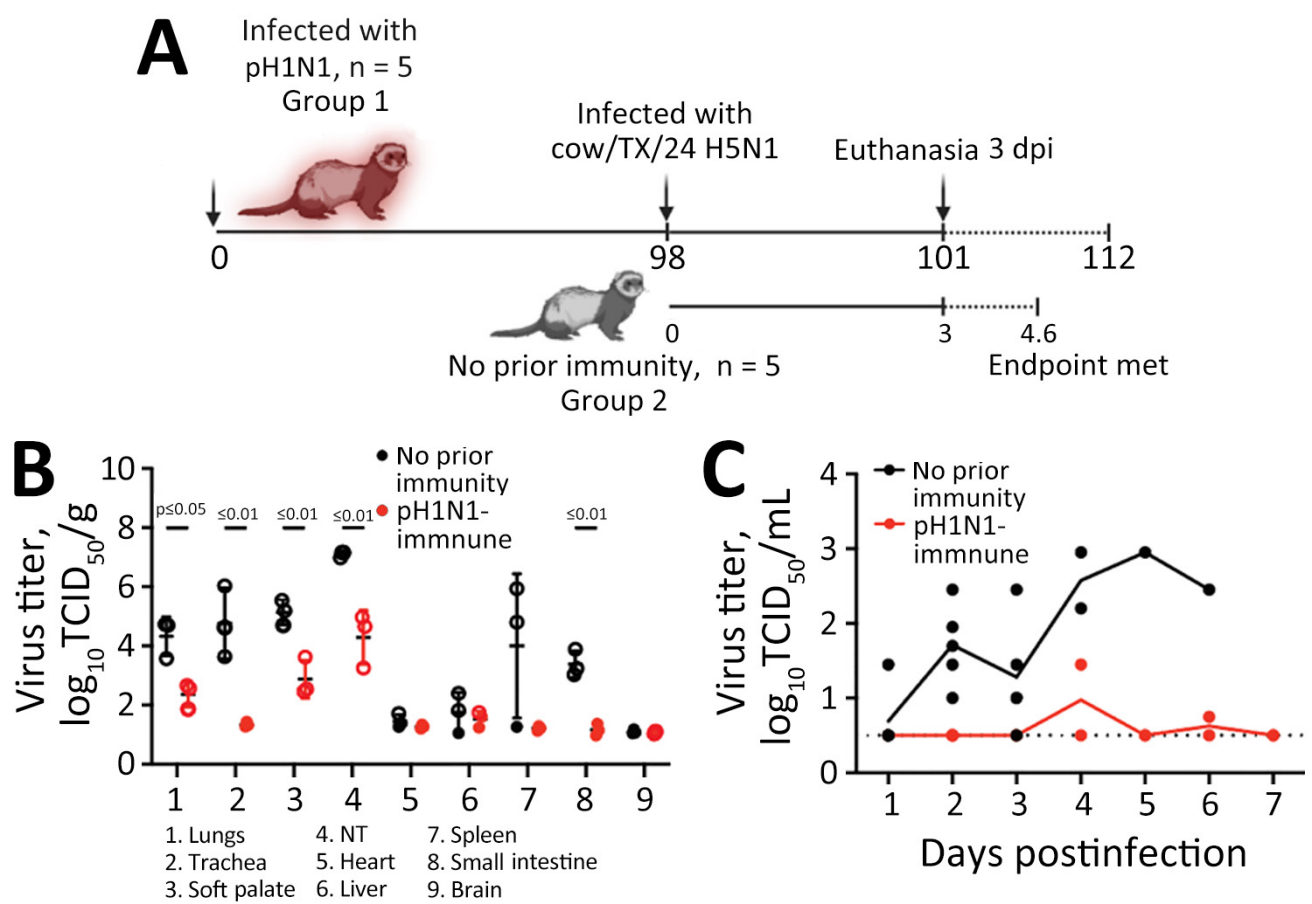
Infection timeline and virus replication titers in a study of influenza A(H5N1) immune response among ferrets with pH1N1 immunity. A) Schematic of experimental timeline for 2 groups of ferrets intranasally infected with H5N1 strain cow/Tx/24. Group 1 (n = 5) was previously infected with pH1N1 98 days before H5N1 infection and group 2 (n = 5) was immunologically naive. At 3 dpi, 3 animals from each group were humanely euthanized. We monitored the remaining ferrets from groups 1 and 2 until 14 dpi or until the endpoint criteria were reached. Schematic was created in BioRender (https://www.biorender.com). B, C) Viral titers from ferret tissues (B) and nasal secretions (C). B) Tissues were collected from H5N1-infected ferrets without (n = 3) and with (n = 3) pH1N1 immunity at 3 days postinfection. Unpaired *t*-test analysis was used to determine statistically significant (p<0.05) differences: lungs, p = 0.0124; trachea, p<0.008; soft palate, p = 0.0072; nasal turbinate, p = 0.0061; small intestine, p = 0.0014. C) Nasal wash samples were collected from H5N1-infected ferrets without (n = 5) and with (n = 5) pH1N1 immunity on the indicated dpi: 1–3 dpi, n = 5 from each group; 4 dpi, n = 2 from each group; 5 and 6 dpi, n = 2 for immune group and n = 1 from the naive group; 7 dpi, n = 2 from the immune group . Each circle represents a single ferret. Open circles indicate values above the limit of detection. Horizontal bars indicate means; whiskers indicate SDs of viral titers; dashed line represents the limit of detection. cow/Tx/24, A/dairy cattle/Texas/24-008749-001/2024(H5N1); dpi, days postinfection; NT, nasal turbinate; pH1N1, influenza A(H1N1)pdm09; TCID_50_, 50% tissue culture infectious dose.

Humane endpoints for this study included bodyweight loss >20% (relative to weight at challenge) and a prolonged inactivity as assessed by trained veterinary staff. Three animals from each group were euthanized 3 days postinoculation (dpi) for tissue titration; the other 2 ferrets from each group were kept for >14 days or until they reached endpoint criteria (Figure 2, panel A). 

### Ferret Sample Collection and Preparation

We collected nasal wash from each ferret at 1–7 days postinoculation (dpi). To examine whether pH1N1 altered cow/Tx/24 H5N1 tissue tropism, we euthanized 3 intranasally infected ferrets from each group at 3 dpi to collect tissues (lungs, trachea, soft palate, nasal turbinates, heart, liver, spleen, small intestine, and brain) and determined virus titers (Appendix).

We titered nasal wash and organ samples in MDCK cell cultures. We made 10-fold serial dilutions and inoculated dilutions on 96-well plates by using 4 wells/dilution. We observed the MDCK cells at 4 dpi for cytopathic effect (CPE). We calculated viral titers by using the Reed and Muench method (*12*) and expressed the results as log_10_ TCID_50_/mL. 

### Animal Ethics Statement

Ferret experiments were conducted in Biosecurity Level 2 and 3 facilities at the University of Pittsburgh in compliance with the guidelines of the Institutional Animal Care and Use Committee (approved protocol nos. 22061230 and 21089461). For all nasal washes and survival blood draws, animals were sedated with isoflurane following approved methods. Ketamine and xylazine were used for sedation for all terminal procedures, followed by cardiac administration of euthanasia solution. Approved University of Pittsburgh Division of Laboratory Animal Resources staff administered euthanasia. H5N1 studies were performed in accordance with the University of Pittsburgh select agent permit no. 20230320-074008.

### Microneutralization Assays

We heat inactivated human and ferret serum samples at 56°C for 30 minutes. We determined the titer of neutralizing antibodies by incubating 2-fold serial dilutions of the heat-inactivated serum samples with 10^3.3^ TCID_50_ of influenza virus for 1 hour at room temperature with continuous rocking. We added media with tosyl phenylalanyl chloromethyl ketone–treated trypsin to 96-well plates with confluent MDCKs before adding the virus-serum mixture. After 4 days, we determined the CPE and expressed the neutralizing antibody titer as the reciprocal of the highest dilution of serum required to completely neutralize the infectivity of each virus on MDCK cells. We calculated the concentration of antibody required to neutralize 100 TCID_50_ of virus on the basis of the neutralizing titer dilution divided by the initial dilution factor, multiplied by the antibody concentration.

### Histology

We stained respiratory tissue sections collected from euthanized ferrets with hematoxylin and eosin for histopathologic analysis or influenza A nucleoprotein for immunohistochemistry (Appendix). We initially examined the prepared slides blinded to experimental groups to eliminate observer bias, then by unblinding for figure preparation. We developed an ordinal scoring system to summarize the histopathologic and immunohistochemical findings: 0, not observed; 1 (mild), <10% of parenchyma impacted; 2 (moderate), from 10%–25% of parenchyma affected; and 3 (severe), 25%–50% of parenchyma affected.

### ELISA

We adhered 500 ng of recombinant HA full-length soluble ectodomains or recombinant neuraminidase (NA) to high-capacity binding 96 well-plates (Corning, https://www.corning.com) overnight in phosphate-buffered saline (PBS) at 4°C (Appendix). We then washed the HA- or NA-coated plates with a 0.05% vol/vol PBS-Tween-20 (PBS-T) buffer and then blocked with PBS-T containing 2% bovine serum albumin for 1 hour at room temperature. We removed the blocking solution and added 2-fold dilutions of ferret serum in blocking solution to the wells. We then incubated the plates for 1 hour at room temperature. We removed the primary antibody solution and washed the plates 3 times with PBS-T. We added a secondary Goat Anti-Ferret IgG H&L (HRP) (Abcam, https://www.abcam.com) diluted 1:10,000 in blocking solution to the wells and incubated for 30 minutes at room temperature. We then washed the plates 3 times with PBS-T. We developed the plates by using 150 μL 1-Step TMB Substrate (Thermo Fisher Scientific). After a brief incubation at room temperature, we stopped HRP reactions by adding 100 μL of 4N sulfuric acid solution. We read the plates by using a SpectraMax 340PC384 Microplate Reader (Molecular Devices, https://www.moleculardevices.com) at 450 nm. We performed all measurements in duplicate. We then graphed the average of the 2 measurements for each ferret sample as the mean absorbance at 450 nm by using Prism software version 9.0 (GraphPad, https://www.graphpad.com).

## Results

### Neutralizing Antibody Levels in Humans

H5N1 IAVs have not circulated widely in the human population, and major immunity against those strains likely does not exist. To assess whether any cross-reactive antibodies existed in the human population, we conducted neutralization assays with human serum against cow/Tx/24 H5N1 and pH1N1, and results revealed high levels of circulating antibodies against pH1N1 in persons of all ages (Figure 1). Of note, 12 of the 60 serum samples tested had detectable levels of cross-neutralizing antibodies against cow/Tx/24 H5N1 that were above the limit of detection. Of the 12 serum samples with cross-neutralizing antibodies, 10 were collected from persons born in the 1940s, 1950s, and 1960s and 2 were from persons born after 1970 (Figure 1), which correlates well with H5 cross-reactive antibodies in older persons (*13*). Those data suggests persons born after 1980 could be more susceptible to infection with H5N1 virus from dairy cows. We do not know the ages of persons with documented H5N1 infections since 2022.

### Effect of pH1N1 Immunity on Viral Titers and Dissemination

We sought to extend our prior work (*9*,*10*) and examine the role of pH1N1 immunity on dairy cow H5N1 infection severity and replication in the ferret model. In ferrets without pH1N1 immunity, cow/Tx/24 H5N1 resulted in high viral loads in the respiratory tissues and produced a systemic infection, as observed by virus detection in the heart, liver, spleen, and intestine (Figure 2, panel B). In contrast, ferrets with pH1N1 immunity exhibited lower levels of virus replication that were limited to the respiratory tract and were statistically significant (p<0.01) (Figure 2, panel B). The lack of virus in the brain of ferrets without pH1N1 immunity at 3 dpi is consistent with data reported from other groups (*8*). 

Nasal wash titers were also drastically different between the 2 groups of ferrets. Virus was consistently detected over time in the nasal washes of ferrets without prior immunity, whereas most pH1N1-immune ferrets had no detectable cow/Tx/24 H5N1 virus in nasal washes; the exceptions were 1 ferret at 4 dpi and a different ferret at 6 dpi (Figure 2, panel C). Of note, we detected virus in the nasal turbinates of the pH1N1-immune ferrets euthanized at 3 dpi, despite a lack of virus in the nasal wash; however, virus levels were much lower than among animals without prior immunity (Figure 2, panels B, C). That difference could be attributed to the methods of sample collection. Nasal washes are performed by pushing fluid through 1 nostril and collecting the liquid from the other nostril, which samples the tip of the turbinates. In contrast, the entire nasal turbinate tissue is collected at the time of necropsy and homogenized to collect any released and cell associated viruses; thus, turbinates would be expected to have higher virus levels.

**Figure 3:**
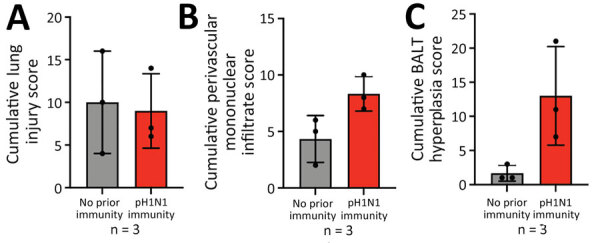
Lung infiltrates measured in a study of influenza A(H5N1) immune response among ferrets with pH1N1 immunity. We blindly scored 5 lung sections for ferrets with no prior or existing pH1N1 immunity for lung injury (A), perivascular mononuclear infiltrates (B), and BALT hyperplasia (C). Each dot represents the cumulative score of the 5 sections for each ferret. Bar values indicate means; whiskers indicate SDs. BALT, bronchus-associated lymphoid tissue; pH1N1, influenza A(H1N1)pdm09.

**Figure 4:**
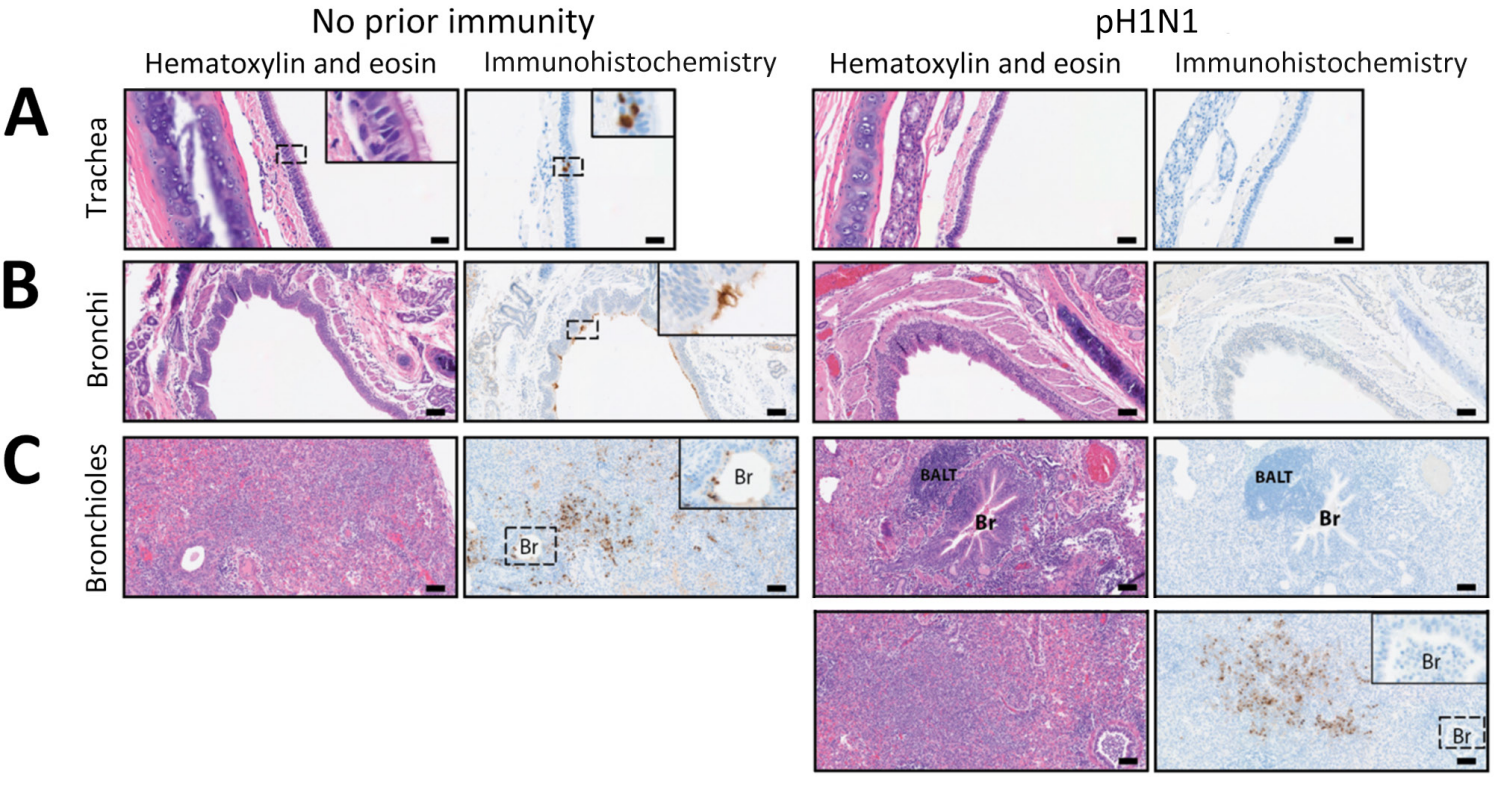
Hematoxylin and eosin–stained and immunohistochemistry tissue samples from a study of influenza A(H5N1) immune response among ferrets with pH1N1 immunity. A) Tracheal tissue. Scale bars indicate 20 mm; inset shows magnification ×400. B) Bronchial tissue. Scale bars indicate 50 mm; inset shows magnification ×200. C) Bronchiole tissue. Scale bars indicate 50 mm; inset shows magnification ×200. Ferrets with no prior immunity (left panels) or existing influenza A(H1N1)pdm09 immunity (right panels) were infected with 10^4^ 50% tissue culture infectious dose of H5N1 strain A/dairy cattle/Texas/24-008749-001/2024(H5N1) and humanely euthanized 3 days postinfection. Images show hematoxylin and eosin stained (purple) tissues and immunohistochemistry of influenza A nucleoprotein (blue). Dotted squares indicate areas that are magnified within the inset panel in tissues from ferrets with no prior immunity versus pH1N1-immune ferrets. BALT, bronchus-associated lymphoid tissue; Br, bronchiole; pH1N1, influenza A(H1N1)pdm09.

Histopathological analysis of lung tissues harvested at 3 dpi indicated both groups of ferrets had similar lung injury (Figure 3, panel A). However, more detailed examination of the data indicated that ferrets with pH1N1 immunity had more residual mononuclear perivascular infiltrates and bronchus-associated lymphoid tissue (BALT) hyperplasia (Figure 3, panels B, C), which may play a role in preventing development of severe clinical disease. Immunohistochemistry with IAV nucleoprotein (NP) indicated that pH1N1-immune ferrets had limited NP-positive cells in the trachea, mainstem bronchi, and bronchioles than ferrets without prior immunity (Figure 4). In pH1N1-immune ferrets with areas of BALT hyperplasia, we detected limited viral antigen and necrotizing bronchointerstitial pneumonia (Figure 4, panel C). Furthermore, we observed IAV NP antigen in alveolar pneumocytes (both type 1 and 2) in ferrets irrespective of immune status (Figure 4, panel C). Those findings are in contrast to pH1N1 infection in ferrets, in which pH1N1 virus infected epithelial cells in the large and small airways (*14*–*18*). 

**Figure 5:**
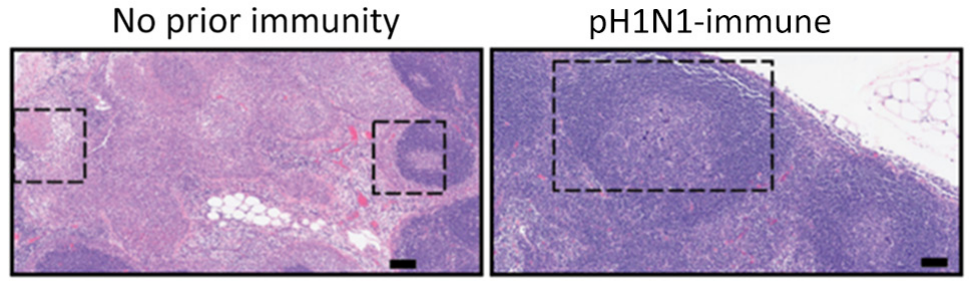
Hematoxylin and eosin–stained lymph node samples from a study of influenza A(H5N1) immune response among ferrets with pH1N1 immunity. Ferrets with no prior immunity (left panel) or pH1N1 (right panel) were infected with 10^4^ 50% tissue culture infectious dose of H5N1 strain A/dairy cattle/Texas/24-008749-001/2024(H5N1) and humanely euthanized 3 days postinfection. Dotted squares indicate areas that are with cortical lymphoid necrosis and depletion in tissues from ferrets with no prior immunity versus normal secondary germinal center in pH1N1-immune ferret. Scale bars indicate 100 mm. pH1N1, influenza A(H1N1)pdm09.

Examination of the tracheobronchial lymph node histology revealed more lymphoid depletion, necrosis, fibrin, and edema in ferrets without pH1N1 immunity compared with pH1N1-immune ferrets (Figure 5). Overall, those data indicated that resident lymphoid changes in ferrets with pH1N1 immunity may have reduced cow/Tx/24 H5N1 replication and dissemination to other organs, which could affect disease severity.

### Effects of pH1N1 Immunity on H5N1 Mortality and Severe Disease 

**Figure 6:**
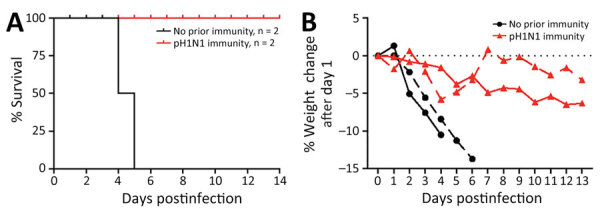
Mortality rates and weight change in a study of influenza A(H5N1) immune response among ferrets with pH1N1 immunity. A) Mortality rates; B) percentage weight change. We observed percentage of weight change as an indicator of severe disease in ferrets with or without pH1N1 immunity among ferrets intranasally infected with H5N1 strain A/dairy cattle/Texas/24-008749-001/2024(H5N1). pH1N1, influenza A(H1N1)pdm09.

**Figure 7:**
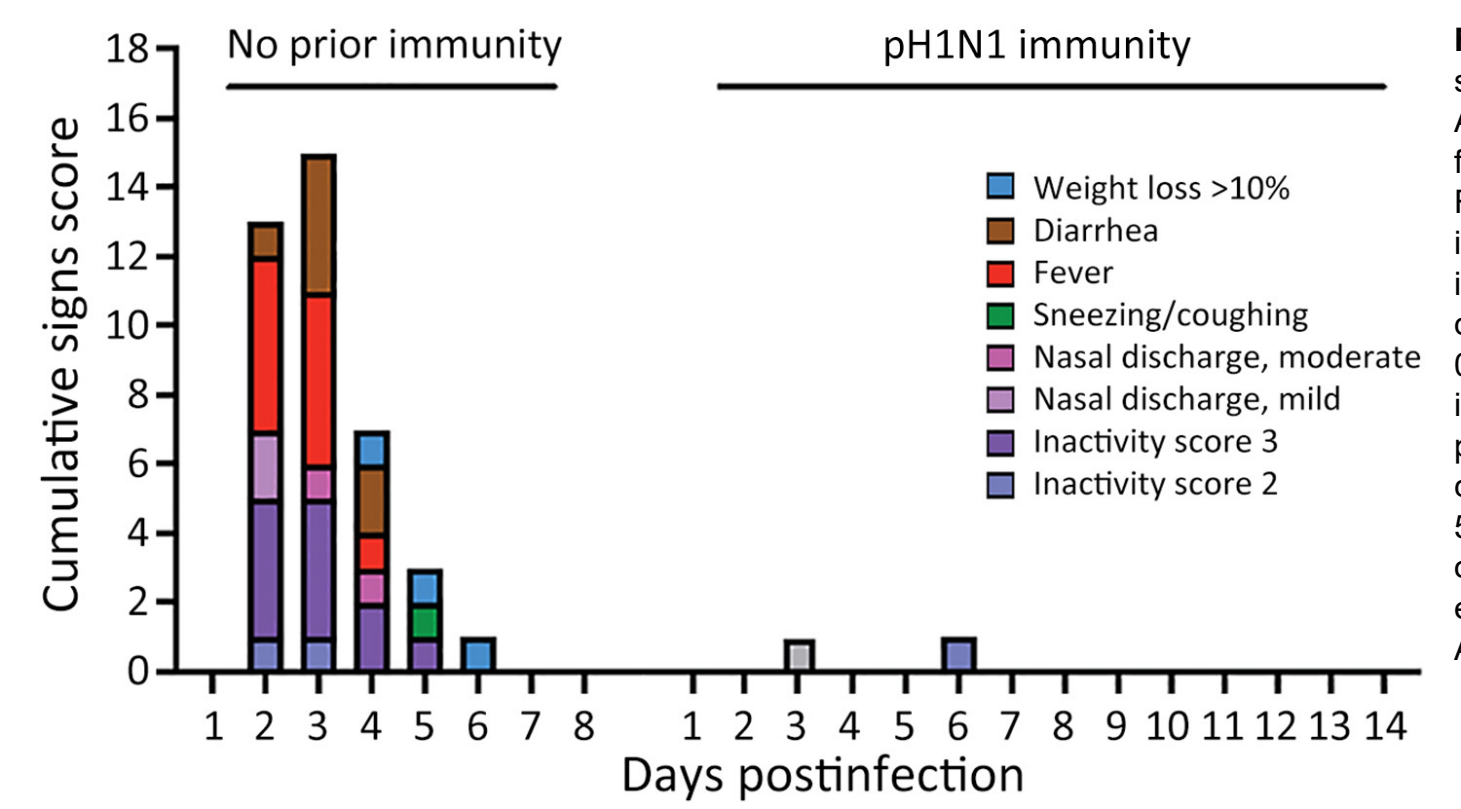
Cumulative clinical signs scores in a study of influenza A(H5N1) immune response among ferrets with pH1N1 immunity. Ferrets with or without pH1N1 immunity were intranasally infected with H5N1 strain A/dairy cattle/Texas/24-008749-001/2024(H5N1). Clinical signs of infection were monitored each day postinfection and quantified into a cumulative signs scores based on 5 ferrets on days 1–3 and 2 ferrets on days 4­–14 postinfection or until euthanasia. pH1N1, influenza A(H1N1)pdm09.

We followed H5N1-infected ferrets with (n = 2) and without (n = 2) pH1N1 immunity to 14 dpi to examine death outcomes (Figure 6, panel A). The 2 animals with pH1N1 immunity survived challenge with cow/Tx/24 H5N1 virus, whereas the 2 immunologically naive ferrets were humanly euthanized at 4 dpi and 6 dpi because severe clinical signs developed (Figure 6, panel A). We observed that ferrets with pH1N1 immunity had <5% weight loss, whereas naive ferrets experienced >10% weight loss (Figure 6, panel B). Assessment of clinical signs, such as diarrhea, fever, nasal discharge and playfulness, revealed more severe clinical signs in all immunologically naive animals than among those with prior pH1N1 immunity (Figure 7). 

Surviving ferrets with pH1N1 immunity seroconverted against cow/Tx/24 H5N1, albeit to low microneutralization titers of 20 and 80 (Table). In addition, neither of the 2 pH1N1-immune ferrets had a >4-fold rise in pH1N1 antibodies after cow/Tx/24 H5N1 challenge (Table). Taken together, those data indicated that pH1N1 immunity protects ferrets from severe clinical disease and death caused by cow/Tx/24 H5N1 infection.

**Table:** Serologic testing from a study of influenza A(H5N1) immune response among ferrets with pH1N1 immunity*

Ferret no.	Euthanasia, dpi	Microneutralization titers					
		H5N1			pH1N1		
		0 dpi	At euthanasia		0 dpi	At euthanasia	
Naive group							
1	3	<20	<20		ND	ND	
2	3	<20	<20		ND	ND	
3	3	<20	<20		ND	ND	
4	4	<20	<20		ND	ND	
5	6	<20	<20		ND	ND	
pH1N1-immune group							
1	3	<20	<20		905	1,016	
2	3	<20	<20		508	320	
3	3	<20	<20		2,032	508	
4	14	<20	20		2,560	2,032	
5	14	<20	80		640	2,032	

*A/dairy cattle/Texas/24-008749-001/2024(H5N1) strain (GISAID accession no. EPI_ISL_19014384) was used for H5N1 inoculation. dpi, days postinoculation; ND, not done; pH1N1, influenza A(H1N1)pdm09.

### Cross-Reactive NA Antibody Production 

**Figure 8:**
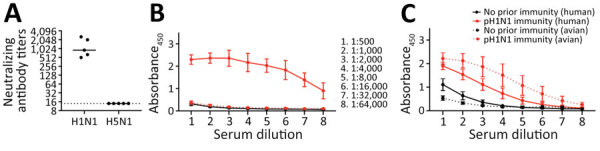
Cross-reactive NA binding antibodies in a study of influenza A(H5N1) immune response among ferrets with pH1N1. Ferrets with or without pH1N1 immunity were intranasally infected with H5N1 strain A/dairy cattle/Texas/24-008749-001/2024(H5N1). A) Serum samples were collected from 5 ferrets with pH1N1 immunity on day 98 postinfection and tested for neutralizing antibodies against pH1N1 and 2024 cow/Tx/24 H5N1 viruses. Each dot represents the antibody titer of a single ferret to neutralize 100 TCID_50_ of pH1N1 or cow/Tx/24 H5N1 on MDCK cells. Solid line indicates the geometric mean value for each virus; dotted line represents the limit of detection for the assay. B) Serum IgG against purified HA proteins in ferrets with or without pH1N1 immunity. Solid lines show ferret serum reactivity to human HA (A/Michigan/45/2015 H1N1) and the dashed lines show ferret serum reactivity to dairy cow HA from A/dairy cattle/Texas/24008749001/2024(H5N1). Dots indicate means; whiskers indicate SDs. C) Serum IgG antibodies against purified NA proteins in ferrets with or without pH1N1 immunity. Solid lines show ferret serum reactivity to human NA (A/California/07/2009 H1N1); dashed lines show ferret serum reactivity to avian NA from A/mallard/New York/22–008760–007-original/2022(H5N1). Dots indicate means; whiskers indicate SDs. Absorbance_450_, absorbance at 450 nm for each dilution; HA, hemagglutinin; NA, neuraminidase; pH1N1, influenza A(H1N1)pdm09; TCID_50_, 50% tissue culture infectious dose.

IAV infection induces antibody responses against HA and NA proteins that can provide varying levels of protection against subsequent infections (*19*). In addition, cross-reactive HA stalk-specific antibodies are able to play a role in reducing influenza virus disease severity (*20*–*22*). To identify immune factors that contribute to the protection of pH1N1-immune ferrets from severe disease, we measured neutralizing and total HA binding antibodies. Before challenge with cow/Tx/24 H5N1 virus, ferrets with pH1N1 immunity exhibited high levels of neutralizing antibodies against pH1N1 but no neutralizing antibodies above the limit of detection against cow/Tx/24 H5N1 (Figure 8, panel A). 

To explore the production of nonneutralizing cross-reactive HA antibodies, we performed an ELISA with serum from ferrets with pH1N1 immunity by using the whole H1 (A/California/07/2009 H1N1) or H5 (A/dairy cattle/Texas/24008749001/2024 H5N1) HA protein (Figure 8, panel B). Ferrets with pH1N1 immunity produced antibodies that bound to H1 as expected but displayed the same background levels of antibody binding to the H5 HA protein as ferrets with no prior immunity (Figure 8, panel B), indicating no detectable cross-reactive HA antibodies against the avian H5 protein. 

Finally, we performed an ELISA using NA from a human (A/Michigan/45/2015 H1N1) or avian (A/mallard/New York/22-008760–007-original/2022 H5N1, which is 98.7% similar to cow/Tx/24 NA) IAV to determine whether pH1N1-immune ferrets had any cross-reacting NA antibodies that might contribute to the protection against severe disease before challenge with H5N1. Of note, serum samples from pH1N1-immune ferrets had antibodies that bound to both the human and avian NA antigens, but ferrets without immunity had background binding levels (Figure 8, panel C). Those data suggest that cross-reactive NA antibodies to avian N1 may be produced from a human seasonal pH1N1 infection.

## Discussion

Influenza A(H1N1)pdm09 immunity in ferrets was sufficient to protect from severe disease and death from highly pathogenic avian influenza A(H5N1) virus from dairy cows. We also observed significantly reduced H5N1 viral titers in nasal secretions and respiratory tract tissues in the animals with pH1N1 immunity (p<0.01). Of note, protection from H5N1 infection was not due to cross-neutralizing antibodies in serum because ferrets with pH1N1 immunity did not generate systemic antibodies that cross-neutralized the cow/Tx/24 H5N1 virus (Figure 8, panel A). Rather, we found ferrets with pH1N1 immunity produced cross-reacting antibodies to H5N1 NA protein (Figure 8, panel C), which is consistent with observations reported from human serologic data (*23*). Immunity to NA has previously been implicated in providing protection during the 1968 H3N2 pandemic (*24*,*25*), and can reduce disease severity of naturally infected and experimentally challenged persons (*26*).

NA antibodies may be involved in protection from severe disease observed in the pH1N1-immune ferrets. However, further studies on the mechanisms of protection are clearly warranted and should include an examination of mucosal immunity from antibodies in the respiratory tract that have broad binding potential. Tissue-resident memory T cells may also help reduce the severity of disease, as is suspected in the case of H1 immunity protecting from airborne transmission of human seasonal H3N2 virus (*9*). A conservation of immunodominant T-cell epitopes between H5N1 and seasonal influenza viruses, including H1N1, was recently reported and suggested to potentially provide a level of cross-protective immunity (*27*). We did note that the lung tissues of ferrets with pH1N1 immunity had increased mononuclear perivascular infiltrates and BALT hyperplasia, consistent with tissue-specific T-cell responses, although additional investigation is required.

The mild infection noted in the 2 pH1N1-immune ferrets that survived until day 14 might account for the low levels of neutralizing antibodies against cow/Tx/24 H5N1 (Table). That observation may be critical to inform the use of H5 seroconversion as a detection mechanism for prevalence of H5 infections in farm workers because mild infections may not produce a robust systemic antibody immune response.

All adults have immunity from repeated influenza virus infections over their lifetimes, but how previous exposures translate into protection may be strain-dependent and change over time. Human H5N1 infections during 2003 had a 30%–50% mortality rate worldwide (*28*). However, since the emergence of the 2.3.4.4b clade in 2020, the mortality rate has been declining; during 2020–2024, at least 80 human infections with various H5N1 clades were reported, but only 8 deaths were reported (*28*). Of note, in 2024, at least 72 human infections and 2 deaths from H5N1 have occurred; both deaths were reported in Cambodia from 2.3.2.1c clade, which is distinct from 2.3.4.4b (*28*). The mild clinical manifestations of H5N1 human cases in the United States could be due to several factors, including changes in the viral genome that result in a less pathogenic virus, inoculation routes and doses, immunity to H1N1 strains that circulated widely since 2010, or a combination of those factors. However, additional research into the level of protection afforded by other human seasonal influenza viruses, particularly currently circulating H1N1 viruses and those before the 2009 H1N1 pandemic, is needed to assess whether currently circulating H1N1 viruses produce a protective immune signature but other prior strains do not. In particular, understanding whether infection from H1N1 or H2N2 strains circulating before 1970 can produce antibodies that cross-react with H5N1 would be useful, given the presence of cross-neutralizing antibodies observed persons born before 1970. Finally, determining whether immunity to conserved regions of the NA or other viral proteins are driving the protection observed with pH1N1 infection is crucial because we detected no neutralizing antibodies in younger persons (Figure 1).

One limitation of this study is the small number of human serum samples used to test for cross-neutralizing antibodies against dairy cow H5N1. However, another group reported similar findings from a different assay (T.A. Garretson et al., unpub. data, https://doi.org/10.1101/2024.10.31.24316514). Other limitations that should be addressed in future work include the small number of animals used in our preimmune studies, use of only 1 subtype of human seasonal virus for the immune imprint, and challenge with only 1 strain of H5N1. Lethality after highly pathogenic avian A(H5N1) influenza infection can be strain specific, and published work has shown that another dairy cattle H5N1 strain was only partially lethal in immunologically naive ferrets (*30*), but a human H5N1 isolate from Texas was lethal in ferrets (*29*,*30*). Therefore, assessment of additional H5N1 strains isolated from dairy cows and human spillover infections is needed. Other groups have performed studies with ferrets having imprints from vaccination or seasonal human influenza virus infection and shown protection against different H5N1 strains (*31*–*35*; P.H. Brigleb et al., unpub. data, https://doi.org/10.1101/2024.10.23.619695). 

In conclusion, we found ferrets with immunity to pH1N1 virus exhibited reduced H5N1 virus replication and dissemination, had less mortality and fewer disease symptoms from H5N1 infection, and expressed H5N1 cross-reacting antibodies to the NA protein. Those results suggest that immunity to heterotypic influenza viruses may explain the mild symptoms observed during 2024 H5N1 infection of dairy and poultry farm workers. Although human H5N1 infections from the 2024 outbreak resulted in mostly mild illnesses, additional research addressing the effects of prior influenza immunity on the pathogenesis and transmission of H5N1 could shed light on the 2024 outbreak strain and inform pandemic risk plans. 

This article was preprinted at https://www.biorxiv.org/content/10.1101/2024.10.23.619881v1.

Appendix Additional information on influenza A(H5N1) immune response among ferrets with influenza A(H1N1)pdm09 immunity.
