# Supplementary material for: Influenza A(H5N1) Immune Response among Ferrets with Influenza A(H1N1)pdm09 Immunity
Source: Emerg Infect Dis. 2025 Mar;31(3):477–87. doi: 10.3201/eid3103.241485 (PMC11878318; doi:10.3201/eid3103.241485)
Supplement: Appendix — Additional information on influenza A(H5N1) immune response among ferrets with influenza A(H1N1)pdm09 immunity. [file 24-1485-Techapp-s1.pdf]

Article DOI: https://doi.org/10.3201/eid3103.241485

EID cannot ensure accessibility for supplementary materials supplied by authors. Readers who have difficulty accessing supplementary content should contact the authors for assistance.

Influenza A(H5N1) Immune Response among Ferrets with Influenza A(H1N1)pdm09 Immunity

Appendix

Materials and Methods

Virus Rescue from Plasmids Using Cocultured Cells

Reverse genetics plasmids with both a pol I and pol II expression system, based off of pHW system (*1*), were used to generate A/dairy cattle/Texas/24-008749-001/2024(H5N1). The plasmids were synthesized based on sequences deposited in the Global Initiative on Sharing All Influenza Data (GISAID) (accession no. EPI_ISL_19014384), with noncoding regions for each segment determined from consensus alignment of H5N1 strains from the 2.3.4.4b clade viruses. Each plasmid containing the 8 segments of A/dairy cattle/Texas/24008749001/2024 was diluted to a concentration of 100 ng/ml and a total of 500 ng of each gene segment was combined with Opti-MEM up to 100 µL and 5 µLof Lipofectamine 2,000 transfection reagent (Life Technologies, Waltham, MA). The transfection mixture was incubated at room temperature for 25 min and transferred to 293T cells in Opti-MEM complete media (Life Technologies, Waltham, MA) in a 6-well plate. After 24 hours of incubation at 37°C with 5% CO_2, 750,000 MDCK cells were added to the 293T cells. Following another 24-hour incubation, a blind-passage of the rescued virus was performed in MDCK cells (London line obtained from International Reagent Resource catalog #FR-58) in a T75 cm^2 flask containing MEM+L-glutamin+TCPK trypsin. The flask was monitored for cytopathic effect (CPE) for 48 h post-inoculation and the RG A/dairy cattle/Texas/24008749001/2024 was isolated in cell culture media and used without further passage. The virus was not passaged in eggs at any time. No mutations were noted in the virus stock compared to the sequence deposited in GISAID EPI_ISL_19014384.

Ferret Screening

Male ferrets were purchased from Triple F Farms (Sayre, PA, USA). Immunologically naive ferrets had a date of birth (DOB) of 12/18/2023 and A(H1N1)pdm09 immune ferrets had a DOB of 10/09/2023. All ferrets were screened by hemagglutinin inhibition (HAI) for antibodies against circulating influenza A and B viruses. The following antigens were obtained through the International Reagent Resource, Influenza Division, WHO Collaborating Center for Surveillance, Epidemiology and Control of Influenza, Centers for Disease Control and Prevention, Atlanta, GA, USA: 2018–2019 WHO Antigen, Influenza A (H3) Control Antigen (A/Singapore/INFIMH-16–0019/2016), b-propiolactone (BPL)-Inactivated, FR-1606; 2014–2015 WHO Antigen, Influenza A (H1N1)pdm09 Control Antigen (A/California/07/2009 NYMC X-179A), BPL-Inactivated, FR-1184; 2018–2019 WHO Antigen, Influenza B Control Antigen, B/Victoria/2/87-like lineage (B/Colorado/06/2017), BPL-Inactivated, FR-1607; 2015–2016 WHO Antigen, Influenza B Control Antigen, B/Yamagata/16/88-like lineage (B/Phuket/3073/2013), BPL-Inactivated, FR-1403. Ferrets with no prior immunity had weights that ranged from 1.47 to 1.68 kg and A(H1N1)pdm09-immune ferrets ranged from 1.2 to 1.6 kg

Ferret Tissue Collection and Processing

The respiratory tissues were collected from euthanized ferrets aseptically in the following order: entire right middle lung, left cranial lung (a portion equivalent to the right middle lung lobe), one inch of trachea (from the cranial region) cut lengthwise, entire soft palate, and nasal turbinates. Tissue samples were weighed, and Leibovitz's L-15 medium was added to make a 10% (lungs) or 5% (trachea) w/v homogenate. Tissues were dissociated in phosphate-buffered saline (PBS) supplemented with antibiotics and antimycotic using BeadBlaster microtube homogenizer and cell debris was removed by centrifugation at 900 xg for 5 minutes. Influenza virus titers were determined by endpoint $TCID_{50}$ assay. Intact lungs including the distal trachea and tracheabronchial lymph nodes were fixed in 10% neutral buffered formalin for 2 weeks and subsequently processed as formalin fixed paraffin blocks (FFPE) following routine histology processes. Microtomy sections were stained with hematoxylin and eosin (H&E) for histopathologic analysis. Immunohistochemistry (IHC) targeting Influenza A Nucleoprotein (Clone F8L6X, Rb origin; Cell Signaling Technologies) was conducted using a Ventana Discovery Ultra autostainer (Roche, Basel, Switzerland) using a primary concentration of 1:200 and a pre-dilute secondary anti-Rb horseradish peroxidase (HRP) polymer (Vector Laboratories,

Newark, California, USA) developed using 3,3′-diaminobenzidine (DAB) chromogen with hematoxylin counterstain (Roche). H&E and IHC slides were scanned using a PhenoImager whole slide scanner (Akoya Biosciences, Malborough, MA, USA) for figure preparation. Slides were initially examined 'blinded' to experimental groups to eliminate observer bias by a board-certified veterinary pathologist (NAC), followed by unblinding for figure preparation. An ordinal scoring system was developed to summarize the histopathologic and immunohistochemical findings: 0-not observed; 1 (mild), <10% of parenchyma impacted; 2 (moderate) >10%, but <25% of parenchyma impacted; and 3 (severe), >25%, but <50% of parenchyma impacted. Each of the 5 lung lobes from each ferret were scored individually. Histopathologic features documented included bronchointerstitial pneumonia, perivascular infiltrates, foci of bronchus associated lymphoid tissue (BALT) and influenza A virus nucleoprotein IHC. A cumulative lung injury score was developed encompassing the severity of bronchointerstitial pneumonia and influenza A virus nucleoprotein IHC scores. Perivascular inflammation and foci of BALT were excluded from the cumulative lung injury score as they are interpreted to represent heterotypic adaptive immunity in the cohort with prior exposure to H1N1 before H5N1 and thus would falsely elevate lung injury scores if included given the paucity of this phenotype in the cohort without prior influenza A virus immunity to H1N1.

Recombinant HA Expression and Purification

Recombinant HA full-length HA ectodomains (FLsE) were expressed by polyethylenimine (PEI) facilitated, transient transfection of 293F cells. To produce FLsE constructs, synthetic DNA was subcloned into a pVRC8400 vector encoding a T4 fibritin (foldon) trimerization tag and a 6xHis tag. The H5 dairy cattle HA was modified to contain stabilizing mutations (*2*) that improved expression and biochemical behavior. Transfection complexes were prepared in Opti-MEM (GIBCO) and added to cells. Five days post-transfection, cell supernatants were harvested and clarified by low-speed centrifugation. HA was purified by passage over TALON Metal Affinity Resin (Takara) followed by gel filtration chromatography on Superdex 200 (GE Healthcare) In 10 mM tris(hydroxymethyl)aminomethane (tris), 150 mM NaCl at pH 7.5.

Recombinant NA Expression and Purification

Recombinant NA constructs for A/Michigan/45/2015 N1 and A/mallard/New York/22-008760-007-original/2022 N1 were expressed using the baculovirus expression system (*3*). The

constructs were designed to have an N terminal signal peptide, followed by a hexahistidine purification tag, the VASP (vasodilator-stimulated phosphoprotein) tetramerization domain, a thrombin cleavage site, and the N1 globular head domain. The baculoviruses were passaged in Sf9 cells and then used to infect High Five cells for protein expression. Recombinant proteins were purified 72 hours post-infection from the High Five cell culture supernatant using gravity flow affinity chromatography using Ni^{2+}- nitrilotriacetic acid (NTA) agarose (QIAGEN).

References

1. Hoffmann E, Neumann G, Kawaoka Y, Hobom G, Webster RG. A DNA transfection system for generation of influenza A virus from eight plasmids. Proc Natl Acad Sci U S A. 2000;97:6108–13. PubMed https://doi.org/10.1073/pnas.100133697

2. Milder FJ, Jongeneelen M, Ritschel T, Bouchier P, Bisschop IJM, de Man M, et al. Universal stabilization of the influenza hemagglutinin by structure-based redesign of the pH switch regions. Proc Natl Acad Sci U S A. 2022;119:e2115379119. PubMed https://doi.org/10.1073/pnas.2115379119

3. Margine I, Palese P, Krammer F. Expression of functional recombinant hemagglutinin and neuraminidase proteins from the novel H7N9 influenza virus using the baculovirus expression system. J Vis Exp. 2013;81:e51112. PubMed https://doi.org/10.3791/51112-v
